# Stimuli-Responsive Polymer-Clay Nanocomposites under Electric Fields

**DOI:** 10.3390/ma9010052

**Published:** 2016-01-15

**Authors:** Shang Hao Piao, Seung Hyuk Kwon, Hyoung Jin Choi

**Affiliations:** Department of Polymer Science and Engineering, Inha University, Incheon 402-751, Korea; sanghoo1105@inha.edu (S.H.P.); 22141016@inha.edu (S.H.K.)

**Keywords:** polymer, clay, nanocomposite, electrorheological, conducting polymer

## Abstract

This short Feature Article reviews electric stimuli-responsive polymer/clay nanocomposites with respect to their fabrication, physical characteristics and electrorheological (ER) behaviors under applied electric fields when dispersed in oil. Their structural characteristics, morphological features and thermal degradation behavior were examined by X-ray diffraction pattern, scanning electron microscopy and transmission electron microscopy, and thermogravimetric analysis, respectively. Particular focus is given to the electro-responsive ER characteristics of the polymer/clay nanocomposites in terms of the yield stress and viscoelastic properties along with their applications.

## 1. Introduction

Nanoscale materials have attracted considerable interest for their potential technological applications in a range of areas because their chemical and physical characteristics can be altered drastically from a macroscopic bulk to the molecular level. In particular, polymeric materials are being improved by forming polymer nanocomposite systems for desired engineering applications because of their synergistic effects on the molecular nanoscale from both pure polymers and inorganic materials [1,2,3]. Among the various inorganics for nanocomposites, there has been particular interest in polymer/clay nanocomposites consisting of a polymer and clay, in which the clay has been selected conventionally as fillers in polymer compound research and industrial applications [4,5]. Particles form internal interfaces with large specific surfaces in dispersions, making it possible to stabilize different director configurations, in which clay minerals are introduced to the structures, either intercalated or exfoliated, because of their small particle size, simple chemical modification mainly using cationic surfactants, layer expanding capabilities with various treatments, and low cost. On the other hand, it is well known that using molecular or nanoscale strengthening techniques instead of conventional particulate filled composites, polymer nanocomposites have the ability to extend their usage, through which they offer unique characteristics that are drastically different from their bulk counterparts [6,7,8,9]. Note that in conventional polymer/clay composites, the clay particles are simply dispersed as a filler in a polymer matrix, mainly to improve the mechanical and thermal properties, such as the heat-distortion temperature [10]. Nevertheless, the characteristics of a polymer-based nanocomposite immediately after its manufacture may be rather different from those of the same material after conversion to a final useful shape by some processing technique. In the case of polymer/clay nanocomposites, Wang *et al.* [11] reported that for a polypropylene/organic modified montmorillonite (MMT) nanocomposite processed via dynamic packing injection molding, the morphological change in the shear-induced morphology with a core in the center, an oriented zone surrounding the core and a skin layer in the cross-section areas of the samples was observed. Another processing technique of ultrasonication also affects the dispersion of clay in a polymer matrix [12].

Concurrently, polymer nanocomposites with conducting polymers with conjugated repeating units have facilitated the new development of enhanced thermal, electrical and optical properties [13,14], via the appraisal of important parameters, such as electron affinities, ionization potential, band gap, and bandwidths [15,16,17], ensuring their potential engineering adoptability with better mechanical strength, dispersion stability or physical properties than pure conducting polymers [18,19,20,21,22,23,24]. In particular, the electrical conductivity can be controlled more easily, and the thermal or mechanical stability can be improved by the fabrication of the nanocomposites [25].

On the other hand, an electrorheological (ER) fluid is a type of smart and intelligent material that possesses extraordinary transition characteristics from a liquid-like to a solid-like state under an applied external electric field [26,27,28,29,30]. In general, a typical ER fluid is formed by electrically polarizable particles dispersed in a non-conducting medium, such as silicone oil or mineral oil [31,32,33,34]. The particles are dispersed randomly in the medium phase, exhibiting Newtonian fluid-like behavior when the external electric field is absent. On the other hand, when an electric field is applied to the fluid, all particles initially dispersed will become rapidly polarized and attract neighboring particles to form a chain-like structure with a robust dipole-dipole interaction aligned along the applied field. When the electric field strength is increased, the resulting chains agglomerate to produce stronger columns, indicating that the ER fluid is a more-like an elastic solid. During this electrically controllable and immediate process, the rheological property of the ER fluids can generally be expressed using a Bingham fluid model. Compared to the liquid-like state of ER fluids without an electric field, the solid-like state induced by an external electric field is provided with a non-vanishing yield stress, higher shear viscosity and dynamic moduli. Therefore, similar to the case of the magnetorheological (MR) fluids under a magnetic field [35,36,37], the ER fluids have a considerable potential in a variety of engineering applications, such as dampers, torque transducers, optical finishing, and haptic devices [38,39,40].

Various electro-responsive materials, including biopolymers, such as cellulose and starch, inorganic particles, such as titania, zeolite, mesoporous material, and silica, and conducting polymers demonstrate typical ER behaviors, while hybrids of organic/inorganic materials have been introduced because of their enhanced characteristics [25].

This paper reviews the fabrication methods and characteristics of smart polymer/clay nanocomposites used as ER materials prepared from a range of different fabrication methods. The ER performance of the polymer/clay nanocomposites was observed by either optical microscopy or rotational rheometry.

### 1.1. Fabrication of Electro-Responsive Polymer/Clay Nanocomposites

As a simple and interesting way to construct organic-inorganic hybrid systems and provide synergistic properties, which cannot be achieved from individual materials, such as easily controlled conductivity and higher mechanical stability, several methods to prepare electro-responsive polymer/clay nanocomposites have been reported [25,40].

#### 1.1.1. *In-Situ* Chemical Oxidation Polymerization

**Scheme 1:**
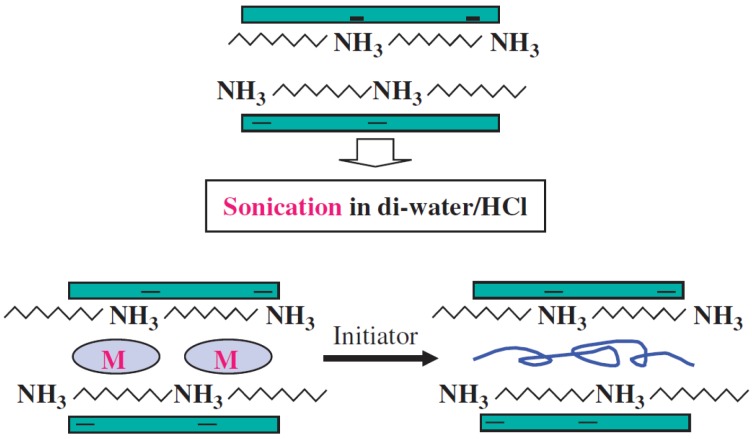
Preparation of the polyaniline (PANI)/clay nanocomposite via an *in-situ* polymerization (Reprinted from Reference [25] with permission).

Yeh *et al.* [41] introduced a polyaniline (PANI)/clay nanocomposite by effectively dispersing the inorganic nanolayers of montmorillonite (MMT) clay in an organic PANI matrix via *in-situ* polymerization. Initially, an appropriate amount of organophilic clay was added to an aqueous HCl solution, and the organic aniline monomers were added to the solution for intercalation into the interlayer regions of the organophilic clay hosts. Upon the addition of ammonium persulfate (APS) in HCl, HCl-doped lamellar nanocomposite precipitates were obtained. The final nanocomposite products were obtained by immersing the HCl-doped nanocomposites into aqueous an NH_4_OH solution, followed by filtration and drying. PANI/clay nanocomposites were prepared in the form of coatings with a low clay loading from 0.75 to 3 wt %. Scheme 1 presents a diagram of the experimental procedure.

Furthermore, using a copolyaniline of poly(o-ethoxyaniline) (PEA), Yeh *et al.* fabricated PEA/MMT nanocomposites with different clay loadings up to 5 wt %, finding that the nanocomposites at low clay loadings up to 3 wt % exhibited a much superior corrosion inhibition effect compared to pristine PEA [42].

Concurrently, using other types of tube-like clay particles, the PANI/halloysite (HNT) nanocomposites have been also reported [43,44,45]. Zhang *et al.* [45] reported the facile synthesis of organic/inorganic PANI-wrapped HNT composite by the *in situ* polymerization of aniline in a HNT dispersion using APS as an initiator, and adopted it as an ER potential material.

**Scheme 2:**
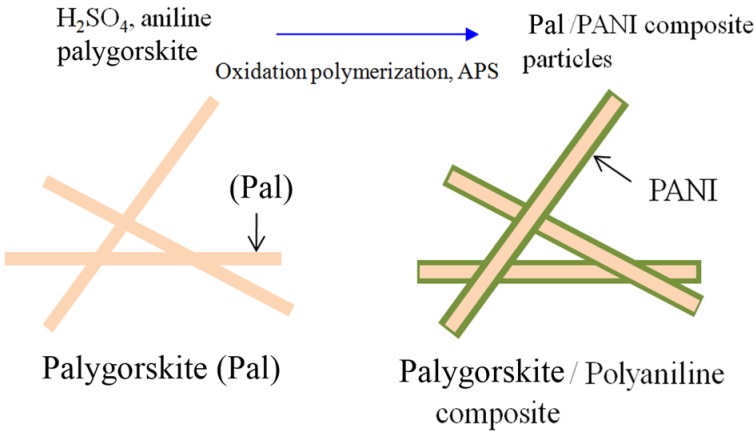
Schematic diagram of the experimental route to synthesize the palygorskite (Pal)/PANI composite particles.

Chea *et al.* [46] fabricated palygorskite (Pal) clay coated with semiconducting PANI nanocomposite particles by an oxidative polymerization process, as shown in Scheme 2 and used them as ER materials without a post-treatment step except for the doping process. Sulfuric acid was added drop-wise to initiate aniline polymerization. The sulfuric acid-modified aniline monomer was then polymerized with the aid of APS at the surface of Pal, resulting in Pal/PANI composite particles.

#### 1.1.2. Suspension Polymerization

Compared to other polymerization processes in electro-responsive polymer/clay nanocomposites, suspension polymerization was seldom adopted. Jun and Suh [47] synthesized poly(urethane acrylate) (PUA)/clay nanocomposite particles by suspension polymerization, with a continuous phase comprised of an aqueous solution of poly(vinyl alcohol) and sodium nitrite, and the dispersed phase comprised of monomer, toluene as an organic diluent, and the oil-soluble initiator with a clay content of 5 wt %. They then examined their ER characteristics.

#### 1.1.3. Emulsion Polymerization

The emulsion polymerization process is considered to be a very useful fabrication technique for synthesizing polymer/clay nanocomposites using glassy polymers with high glass-transition temperatures, such as poly(methyl methacrylate) (PMMA), polystyrene (PS) and styrene-acrylonitrile (SAN), and epoxy and rubbery polymers, such as poly(ethyl acrylate) [25,48]. Kim *et al.* [49] synthesized SAN copolymer-Na^+^-MMT clay nanocomposite particles by emulsion polymerization and examined their ER performance. For the emulsion polymerization of polymer nanocomposites, the Na^+^-MMT clay was introduced to synthesize a polymer/clay nanocomposite with SAN in the presence of potassium persulfate as an initiator and sodium lauryl sulfate as an emulsifier, and the product was then coagulated by the addition of aluminum sulfate solution. The final weight fraction of the Na^+^-MMT in the SAN/clay nanocomposite was 4.76 wt %. ER fluids composed of SAN-clay composite exhibited typical ER behavior and possessed “pseudo-Newtonian” behavior at high shear rates.

#### 1.1.4. Pickering Emulsion Polymerization

Recently, the Pickering emulsion polymerization process has attracted considerable attention as a new method for the fabrication of smart nanocomposites, in which the emulsion droplets prior to polymerization are being stabilized by various solid particles instead of conventional organic surfactants or stabilizers. Therefore, Pickering emulsions impart better stability against coalescence and, in many cases, are biologically compatible and environmentally friendly [50]. Although Pickering emulsions have huge industrial potential applications in the areas of petroleum, food, biomedicine, pharmaceuticals, and cosmetics, Pickering emulsion polymerized particles have recently been adopted for both ER and MR materials [51].

Fang *et al.* [52] fabricated PANI/clay nanoparticles with a special core-shell structure via Pickering emulsion in a toluene phase by employing an exfoliated clay sheet as a stabilizer, using organophilically modified MMT (OMMT). The synthesized PANI nanospheres, which were initialized by oil-soluble benzoyl peroxide, possessed a polydisperse size distribution of particles, ranging from 200 nm to 1 μm.

**Scheme 3:**
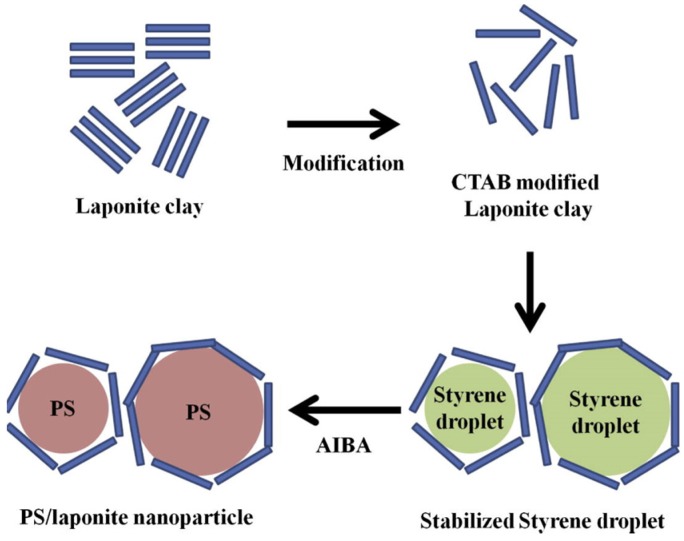
Mechanism of surfactant-free Pickering emulsion polymerization for polystyrene (PS)/laponite nanoparticles (Reprinted from Reference [53] with permission).

Kim *et al.* [53] introduced polystyrene (PS)/laponite composite nanoparticles fabricated using Pickering emulsion polymerization. The hydrophilic laponite modified with cetyltrimethylammonium bromide was used as a stabilizer, in which emulsions of styrene were dispersed in water. Scheme 3 outlines the mechanism for preparing PS/laponite core-shell particles by surfactant-free Pickering emulsion polymerization. The modified laponite was adsorbed on the surface of the styrene monomer droplet to stabilize the system. After adding a water soluble initiator, the mixture became milky white and polymerization occurred in the styrene droplets with laponite adsorbed at the boundary surface. The weight ratio of laponite in the PS/laponite nanoparticles was approximately 8.85%.

### 1.2. Melt Processing

**Scheme 4:**
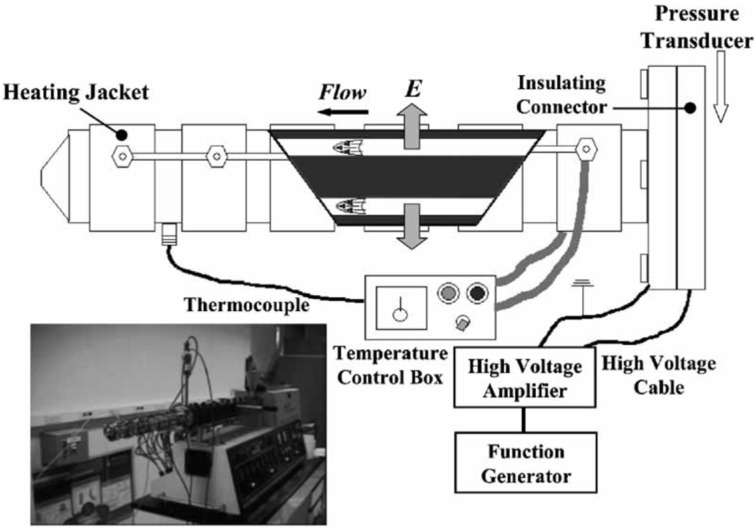
Schematic diagram of an electric melt pipe equipped with a twin-screw extruder. (Reprinted from Reference [55] with permission).

Based on the effects of the applied electric field on the structural evolution of poly(propylene) (PP)/clay nanocomposites, showing a tendency toward exfoliation [54], Kim *et al.* [55] reported a novel method to produce poly(propylene)/clay nanocomposites continuously with a clay content of 5 wt % using an electric melt pipe equipped with a twin-screw extruder, as shown in Scheme 4. In their study, partial intercalation was obtained by continuous processing, showing the possibility to produce nanocomposites using this method. As this physical process can be appropriate for conventional extrusion, the approach may also be used in other polymer/clay nanocomposite systems [55].

## 2. Characterization of Polymer-Clay Nanocomposites

### 2.1. Morphology

Once the electro-responsive smart polymer/clay nanocomposites were synthesized, their morphology was examined to determine if the nanocomposite had been synthesized successfully. These can be answered by either scanning electron microscopy (SEM) or transmission electron microscopy (TEM).

**Figure 1:**
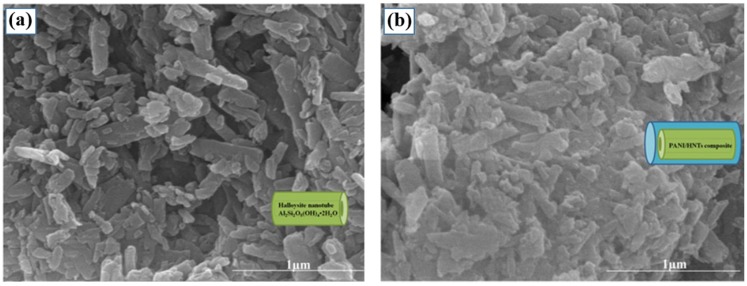
Scanning electron microscopy (SEM) images of (**a**) halloysites (HNTs) and (**b**) PANI/HNT composites (Reprinted from Reference [45] with permission).

Figure 1 provides direct evidence of the synthesis of a PANI-coated HNT surface provided by the SEM images, in which the neat nanotubes of the raw HNT can be seen. Compared to the pure HNT, the distinctive tubular shape of the PANI/HNT composite in Figure 1b disappeared, indicating the formation of PANI.

**Figure 2:**
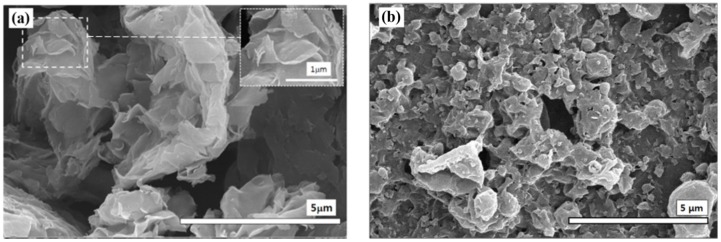
SEM images of pristine clay (**a**) and fabricated PANI/OMMT nanocomposite particles (**b**) (Reprinted from Reference [52] with permission).

Regarding the morphology of the PANI/OMMT nanocomposite confirmed by SEM (Figure 2a), pure clay exhibits a lamellar structure with a nanosized thickness and huge surface area in each layer. After the polymerization of aniline, a large number of nano-granules were observed with a broad particle size distribution, as shown in Figure 2b. The granular surface became rough due to the adsorbed OMMT sheets [56,57,58,59]. Therefore, the PANI/OMMT nanocomposite particles have a core-shell structure, in which the core material is an organic PANI and the shell material is the exfoliated clay sheet.

**Figure 3:**
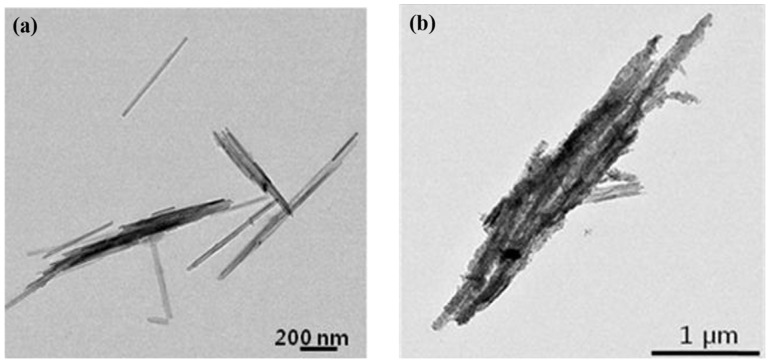
Transmission electron microscopy (TEM) images of Pal (**a**) and Pal/PANI composite particles (**b**), respectively (Reprinted from Reference [46] with permission).

The morphologies of both Pal/PANI and Pal were examined by TEM to characterize the surface morphology of the pure Pal (Figure 3a) and Pal/PANI particles (Figure 3b). Figure 3a showed that the surface of Pal was quite smooth, in which Pal has a highly fibrous morphology that forms bundles. The length of each fiber was varied from the sub-micrometer to micrometer range with a mean diameter of approximately 20 nm. In contrast, the Pal/PANI composite particles had a much rougher surface due to the wrapping of PANI (Figure 3b), meaning that the aniline had been polymerized onto the Pal template by a chemical oxidation method, altering the outside surface.

**Figure 4:**
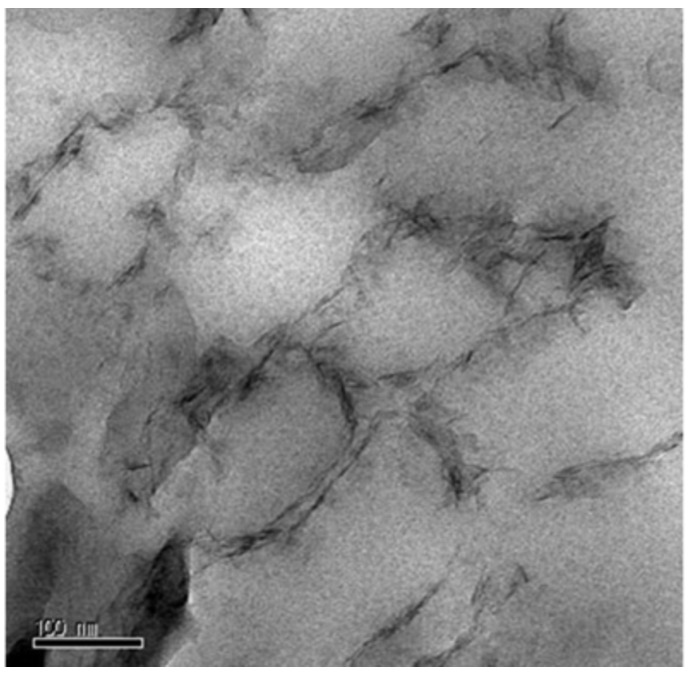
TEM image of a poly(methyl methacrylate) (PMMA) nanoparticle stabilized by laponite (Reprinted from Reference [60] with permission).

Fang *et al.* [60] published a TEM image of a cross-sectional view of the synthesized nano-sized laponite stabilized poly(methyl methacrylate) (PMMA) spheres, as shown in Figure 4. The PMMA nanospheres were synthesized by surfactant-free Pickering emulsion polymerization, the emulsions of methyl methacrylate (MMA) monomer were dispersed in water stabilized by the hydrophilic laponite clay. The grey spherical regions were considered to be PMMA cores and the dark strips were laponite plates. The grey cores were surrounded by multitudinous densely stacked laponite plates, demonstrating the role of laponite plates as a stabilizer.

### 2.2. Crystalline State

In the case of crystalline clay, the delicate layer-layer structure and the changes in the d-spacing between adjacent layers were examined by X-ray diffraction (XRD). The initial XRD patterns were different from the final XRD patterns, in which the characteristic peak for clay showed a shift in intensity and position.

**Figure 5:**
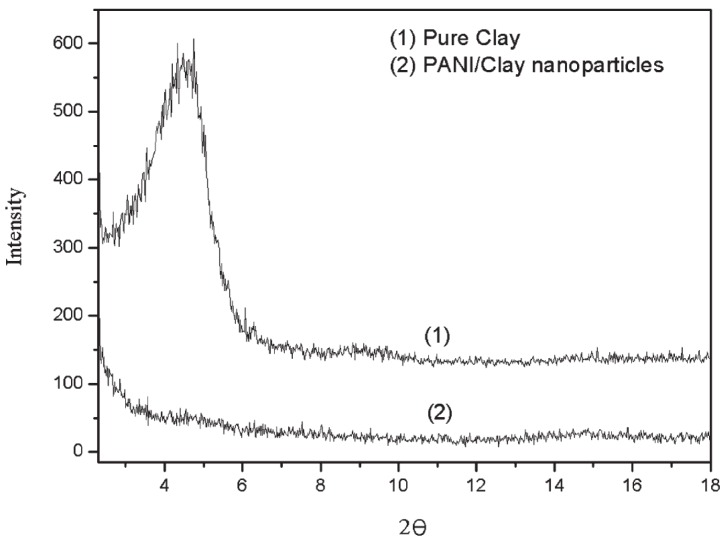
X-ray diffraction (XRD) patterns of pure clay (1) and clay sheet-stabilized polyaniline granules (2) (Reprinted from Reference [61] with permission).

From the XRD pattern of pure clay and PANI/clay nanoparticles [61] (Figure 5), the salient peak (d001) equivalent to the basal spacing of MMT was calculated to be 3.25 nm using the Bragg Equation: λ = 2*d*·sinθ (λ = 0.154 nm) [62]. The PANI/clay nanoparticles in Figure 5 showed no distinct sharp peak but a wide plateau, proving the disappearance of the layered structure. Nevertheless, it can be deduced that a few clay particles were not exfoliated, and the entire exfoliation of clay remains a difficult task [63].

**Figure 6:**
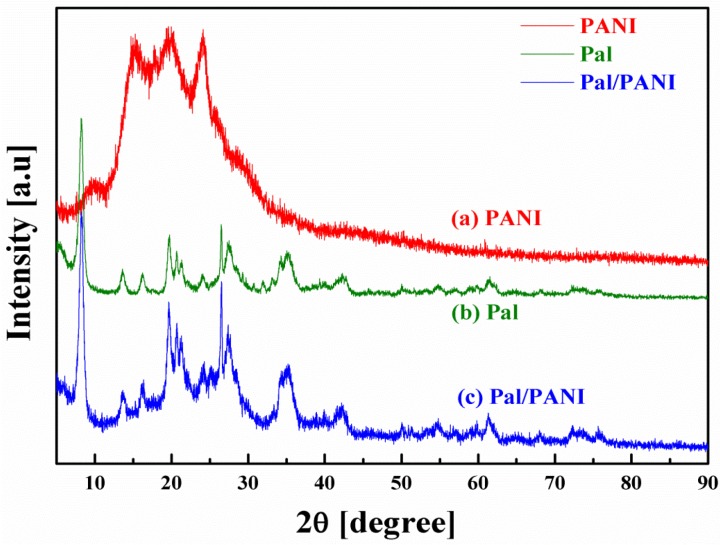
XRD patterns for pristine PANI (**a**), Pal (**b**) and Pal/PANI (**c**) (Reprinted from Reference [46] with permission).

Figure 6 presents XRD patterns of Pal, PANI and synthesized Pal/PANI nanocomposite particles. The crystalline PANI possessed reflections at 18.4° and 25.7° 2θ. The reflection centered at 18.4° 2θ was due to the periodicity in the orientation parallel to the polymer chain, while the peak at 25.7° 2θ was assigned to the periodicity in the orientation perpendicular to the polymer chain [64]. For Pal, the representative reflections were observed at 8.3°, 13.6°, 19.7° and 26.6° 2θ, which were assigned to the (1 1 0), (2 0 0), (0 4 0) and (4 0 0) planes of Pal, respectively [65]. Pal/PANI showed a similar set of characteristic reflections, which means that the crystal structure of Pal has been maintained during its polymerization reaction. The PANI reflection in Pal/PANI is almost invisible because Pal/PANI has a relative thin layer of amorphous PANI synthesized by this polymerization reaction [65].

### 2.3. Thermal Properties

The nanoscopic combination of conducting polymers with clay affects the thermal performance, in which the enhanced thermal properties are due to the significant enhancements in the interfacial conglutination between the polymer and clay [66]. Generally, better interfacial bonding imparts better properties to a polymer nanocomposite, such as tensile strength, hardness and high modulus, as well as resistance to fatigue, tear, corrosion and cracking [67]. Thermogravimetric analysis (TGA) shows the changed thermal behaviors.

**Figure 7:**
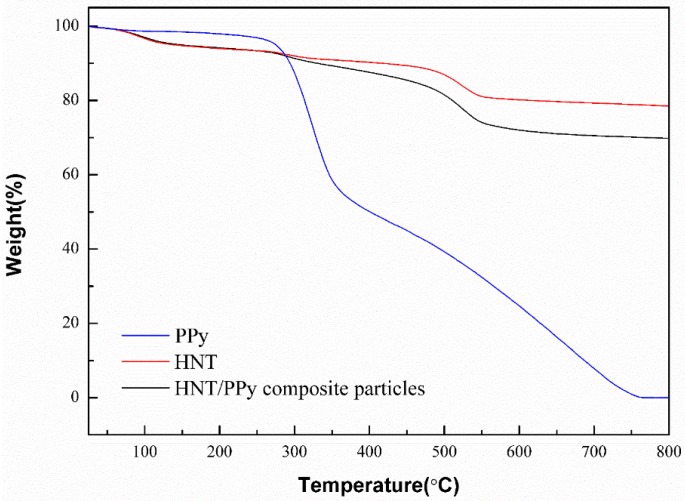
Thermogravimetric analysis (TGA) curve of HNT and PPy/HNT composite (Reprinted from Reference [68] with permission).

Figure 7 presents the weight composition and thermal stability of the HNT and polypyrrole (PPy)/HNT nanocomposite [68]. The PPy/HNT nanocomposites exhibited two-step thermal degradation behavior with the first weight loss from 300 °C and the second thermal degradation from 500 °C, indicating that they were thermally stable up to 300 °C. A sharp loss in mass was observed at 300 and 500 °C, possibly due to the large scale thermal degradation of the PPy chains [69] and the dehydroxylation of HNT [70]. On the other hand, this char formation temperature indicates the increased thermal stability of the polymer/clay nanocomposite [71].

## 3. Electrorheological (ER) Characteristics

**Figure 8:**
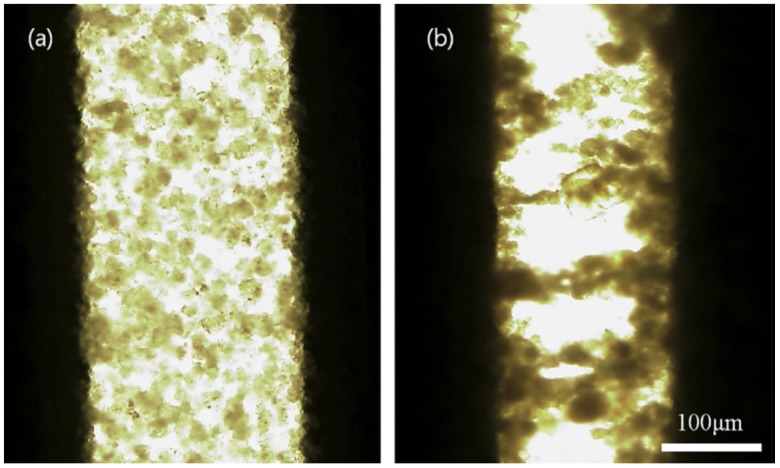
Optical microscope (OM) images of the electrorheological (ER) fluid based on PS/laponite nanoparticles in the absence of an electric field (**a**) and in an electric field (**b**) (Reprinted from Reference [53] with permission).

Kim *et al.* [53] prepared the an ER fluid by dispersing the PS/laponite nanoparticles in silicone oil and observed its structural change directly by optical microscope (OM) under an external electric field with a Direct current (DC) high voltage source. The PS/laponite nanoparticle-based ER fluid exhibited a typical ER chain structure. In the absence of an electric field, the particles were dispersed randomly in silicone oil, indicating a liquid-like state (Figure 8a). In an applied electric field, the particles moved immediately and formed a chain structure aligned along the orientation of the applied electric field (Figure 8b). Normally, this phenomenon of chain formation under an external applied electric field can be maintained as long as the electric field is applied.

**Figure 9:**
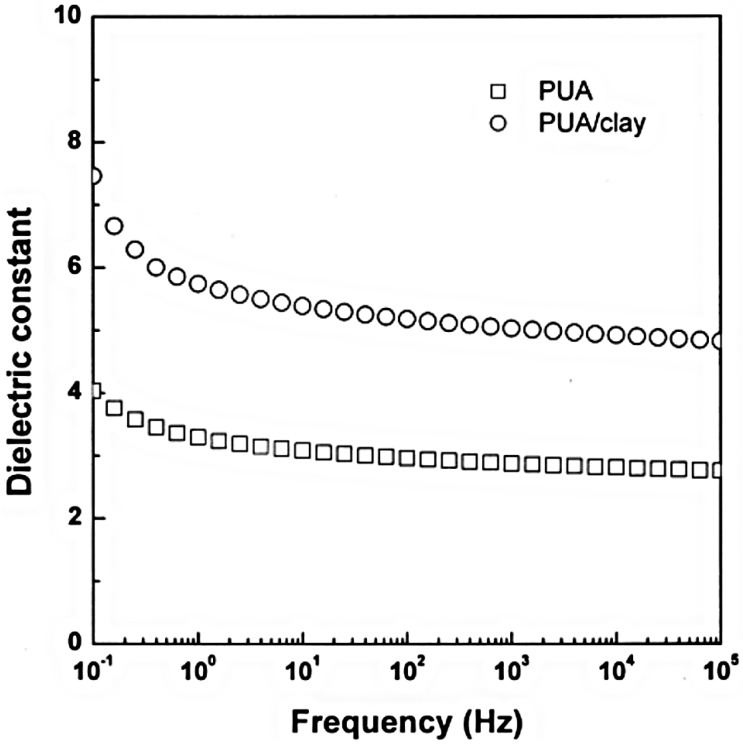
Dielectric constants as a function of the applied electrical frequency for bare poly(urethane acrylate) (PUA) and PUA/clay composite particles (Reprinted from Reference [47] with permission).

Owing to the dielectric polarization [72,73] of ER fluids arising from the mismatch between the dielectric constants of the medium oil and the dispersed nanocomposites, particulate materials with a higher dielectric constant [74], are expected to be beneficial for the superior ER effects. Figure 9 shows the variation of the dielectric constants as a function of the applied electrical frequency for the PUA and PUA/clay composite particles [47]. The clay amalgamated PUA particles showed significantly improved dielectric constant values than the bare PUA particles.

Regarding the flow curve, however, the Pal/PANI nanocomposite particle-based ER fluid exhibited an unusual decreasing trend in shear stress at a low shear rate region, and an increased shear stress with an increasing shear rate. The chain structures began to deteriorate with hydrodynamic shear deformation, and the damaged structures tended to reform the chains repeatedly due to the applied electric field, depending on the magnitude of the applied shear and the particle to particle interactions in the fibrils. In the low shear rate region, the electrostatic interactions were dominant [75,76]. At a high shear rate region, where the hydrodynamic interaction was greater, the broken chain particles had less chance to reform and the ER fluid behaved like a pseudo-Newtonian fluid [75]. Therefore, the Bingham fluid model and Cho–Choi–Jhon (CCJ) model were used to explain the shear stress behavior and yield stress [76].

**Figure 10:**
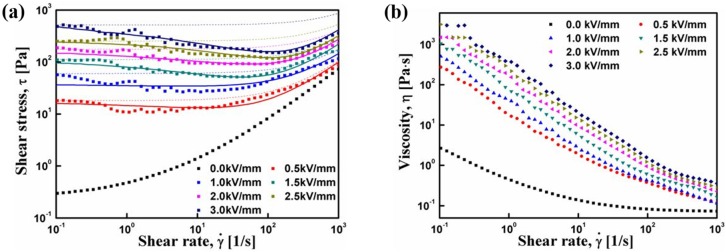
Shear stress (**a**) and shear viscosity (**b**) curves *vs.* shear rate for Pal/PANI based on ER fluids (10 vol. %) under a range of electric field strengths, the dashed lines were fitted using a conventional Bingham model, the solid lines were fitted via a suggested CCJ model (Reprinted from Reference [46] with permission).

The Bingham fluid model shown in Equation (1), which is the simplest model with two parameters originating from Newtonian viscosity (η_0_) and yield stress (τ_0_), is used widely to describe the shear stress behavior of ER suspensions and conventional suspension systems.

(1) $$\begin{array}{c} \tau =\tau_{0}+\eta_{0}\dot{\gamma }\;(\tau \geq \tau_{0})\\ \dot{\gamma }=0\;(\tau <\tau_{0}) \end{array}$$

where τ represents the shear stress and $\dot{\gamma }$ is the shear rate. The dotted lines in Figure 10a were from Equation (1). The simple Bingham model, however, could not be fitted to the flow curve of Pal/PANI ER fluid. Therefore, the CCJ model shown in Equation (2) was suggested to re-plot the shear stress behavior by fitting the curves using six parameters [75].

(2) $$\tau =\frac{\tau_{0}}{1+{\left(t_{1}\dot{\gamma }\right)}^{\alpha }}+\eta_{\infty }\left(1+\frac{1}{{\left(t_{2}\dot{\gamma }\right)}^{\beta }}\right)\dot{\gamma }$$

where η*_∞_* represents the viscosity at the infinite shear rate that is interpreted as the viscosity in the absence of an external electric field. The parameters, *t*_1_ and *t*_2_, are time constants, the exponents α and β are defined as the decrease and increase in shear stress; the exponents β has the range 0 < β ≤ 1, due to $\frac{d\tau }{\dot{\gamma }}\geq 0$ [68]. Figure 10a shows the fitting of the two model equations for the Pal/PANI composite-based ER fluid. The solid lines from the CCJ model showed a better fit to the flow curves than the dotted lines generated by fitting the Bingham model in both the low and high shear rate regions [75].

As shown in Figure 10b, the shear viscosity exhibited shear thinning behavior as a function of the shear rate as similar to that in various polymeric systems [77]. Generally, the non-Newtonian behavior in the absence of an electric field is due to the particle dispersed state in a high concentrated ER fluid.

**Figure 11:**
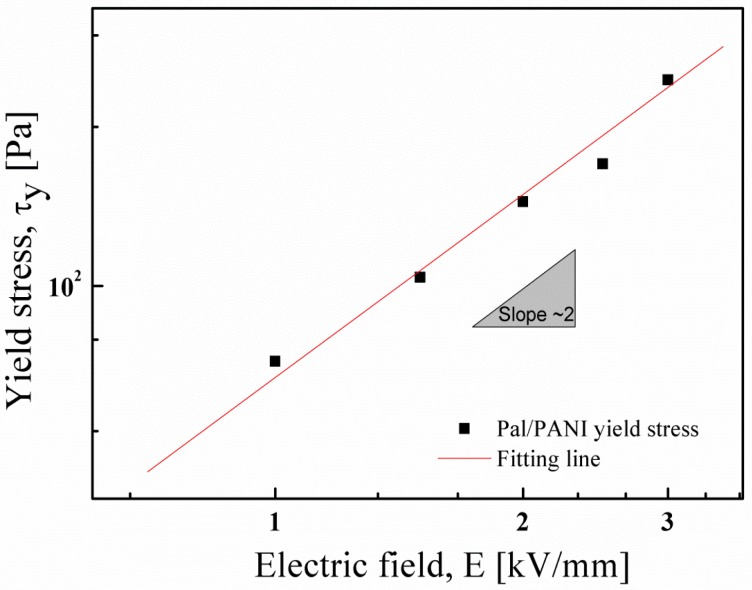
Dynamic yield stress as a function of the electric field strength of the Pal/PANI composite particles based ER fluid. The solid line was fitted using the equation, τ*_y_* ∝ *E^2^* (Reprinted from Reference [46] with permission).

The dynamic yield stress is a characteristic factor of an ER fluid. In Figure 11, the dynamic yield stress of the Pal/PANI composite particle-based ER fluid was plotted as a function of the electric field strength (*E*) in log–log scale curves. The yield stress (τ*_y_*) of an ER fluid is related to the electric field strength, and can be described as a power law relationship:

(3) $$\tau_{y}\propto E^{m}$$

where the exponent *m* was obtained by fitting the yield stress over a broad electric field range. The dependence of the dynamic yield stress could be expressed as τ*_y_* ∝ *E^2^*, which is a polarization model of the ER mechanism [78].

**Figure 12:**
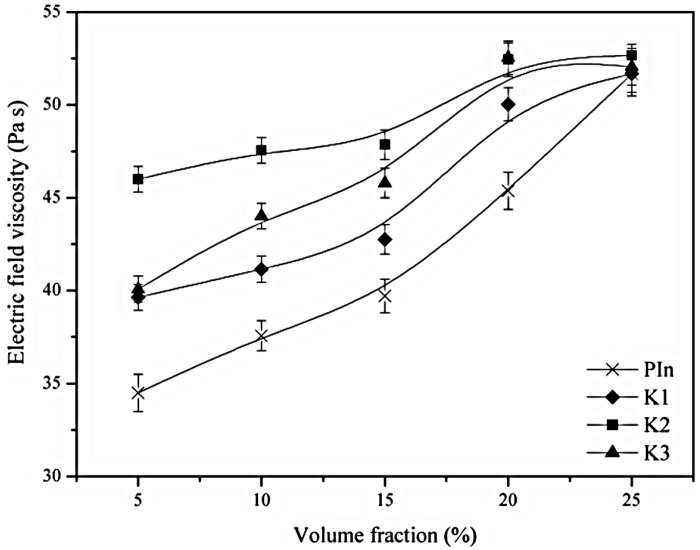
Effect of volume fraction on electric field viscosity (*E* = 3 kV/mm, $\dot{\gamma }$ = 1·s^−1^, *T* = 25 °C). (Reprinted from Reference [81] with permission).

Volume fraction of electro-responsive ER particles dispersed in the suspension is one of the major factors that affect the electric field dependent shear viscosity [79,80,81,82,83]. Guzel *et al.* [81] fabricated polyindene (PIN) and three volume fractions of OMMT nanocomposites namely K1 (5.5%), K2 (7.2%) and K3 (12.1%) to study their ER characteristics. Figure 12 shows the change of the electric field-dependent shear viscosity with clay volume fraction at constant conditions (*E* = 3 kV/mm, $\dot{\gamma }$ = 1·s^−1^, *T* = 25 °C). The higher particle concentration leads to the intensive particle chains formed by the influence of an external electric field, resulting in a higher resistance to flow.

**Figure 13:**
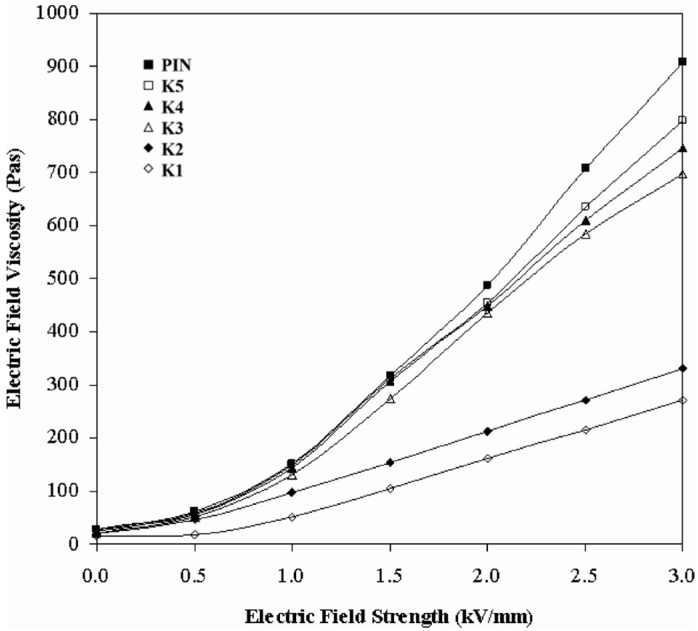
The change of viscosity with electric field strength, (*c* = 15 (m/m, %), $\dot{\gamma }$ = 1.0·s^−1^, *T* = 25 °C). (Reprinted from Reference [80] with permission).

Furthermore, Eristi *et al.* [80] synthesized PIN and five PIN/kaolinite composites containing different amounts of kaolinite of K1 (78%), K2 (63%), K3 (47%), K4 (25%) and K5 (15%). Figure 13 shows that the electric field-dependent shear viscosity increased with enhanced electric field strength. However at a given electric field applied of 3 kV/mm, it was observed that the electric field viscosity reduced with increasing clay content.

**Figure 14:**
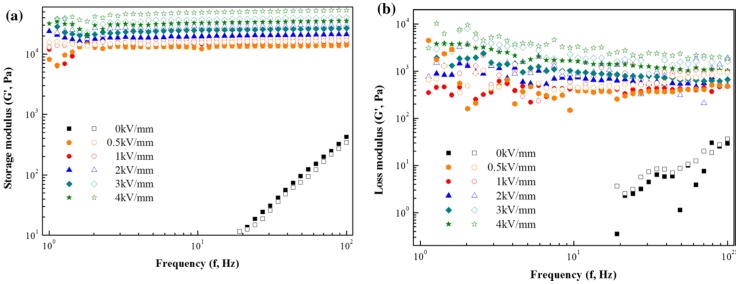
Storage modulus (**a**) and loss modulus (**b**) *versus* frequency for PPy/HNT composite-based ER fluid (close symbol 10 vol. % and open symbol 15 vol. % particle concentration) under various electric field strengths (Reprinted from Reference [68] with permission).

Figure 14 compares the frequency dependence of the storage modulus (*G′*) and loss modulus (*G″*) of a PPy/HNT fluid, the measured frequency range was 1–100 rad/s. In the absence of an electric field, the storage modulus increased linearly with frequency, showing liquid-like characteristics. In an applied external electric field, the *G′* and *G″* values increased in proportion to the electric field. The *G′* values representing an elastic response were higher than those of *G″*, representing viscous property indicating that the ER fluid has very strong solid-like behavior, which is the dominant factor of the elastic property over the viscous one.

**Figure 15:**
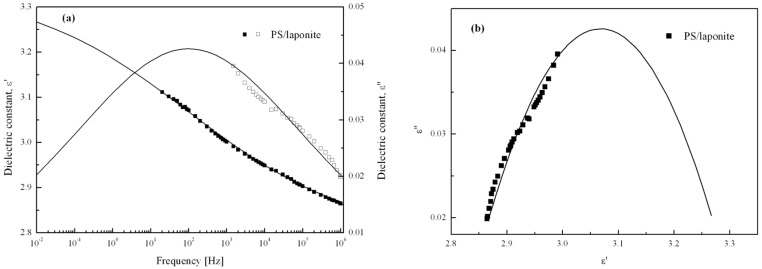
(**a**) Dielectric spectra (ɛ*′*: closed symbols; ɛ*″*: open symbols) and (**b**) Cole–Cole plot of the ER fluids. The fitting lines are generated from Equation (4) (Reprinted from Reference [53] with permission).

To further examine the ER characteristics of the PS/laponite nanoparticle-based ER fluid, the relationship between dielectric and ER properties were examined using an inductance capacitance resistance (LCR) meter. Figure 15 shows the dielectric spectra and Cole–Cole plot, respectively [84]. Both the permittivity (ɛ*′*) and loss factor (ɛ*″*) were measured as a function of frequency (*ω*). The model is represented in terms of the complex dielectric constant as follows:

(4) $$\varepsilon^{*}=\varepsilon^{\prime }-\varepsilon^{\prime \prime }=\varepsilon_{\infty }+\frac{\Delta \varepsilon }{{\left(1+i\omega \lambda \right)}^{1-\alpha }}$$

In Equation (4), ε* is a complex dielectric constant and *ε*_0_ is the dielectric constant when *ω* approaches 0. Δε is the difference between the dielectric constant at 0 and infinite frequency (ε_0_ and ε*_∞_*). They are the distribution curves over a broad frequency range. λ is the dielectric relaxation time at the frequency of which the dielectric loss arrives the maximum value. The exponent (1–α) represents the broadness of the relaxation time distribution and α is a value in the range 0–1. When α is zero, Equation (4) reduces to the Debye’s single relaxation time model. Δε is the achievable polarizability in the ER fluids, which equals 0.546. Owing to the laponite content in the PS/laponite particles, the value of Δε was much lower than those reported elsewhere [85,86,87]. A similar correlation between the dielectric properties and ER performance was also reported for the silica nanoparticle decorated polyaniline nanofiber-based ER fluid [88].

**Figure 16:**
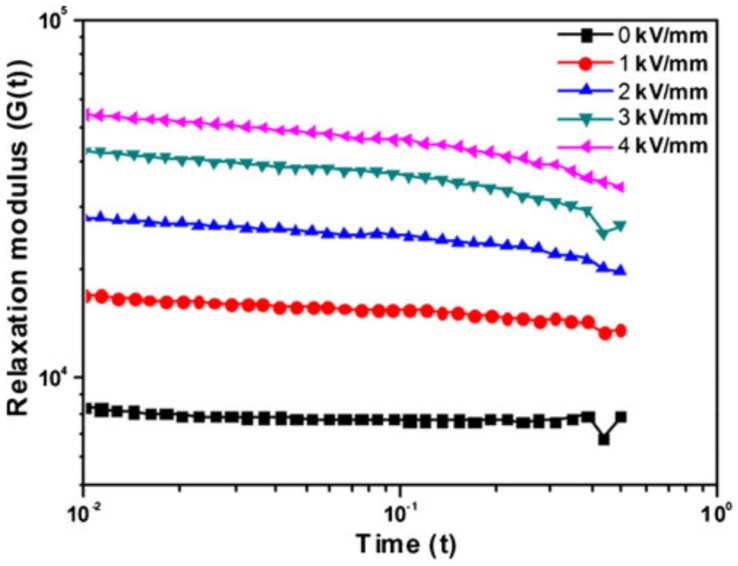
Relaxation modulus *G*(*t*) of PANI/HNT composite-based ER fluid as calculated from *G*′(ω) and *G*″(ω) (Reprinted from Reference [45] with permission).

As shown in Figure 16, observing the relaxation behavior is one way of inspecting the phase change from a liquid-like to solid-like phase. The relaxation modulus *G*(*t*) was calculated from the *G*′ and *G*″ values using the typical formula known as the Schwarzl Equation, as given in Equation (5). Note that *G*(*t*) is difficult to measure experimentally due to the intrinsic properties of the materials and the limitation of the mechanical measurements [89]. *G*(*t*), as a function of time showed a linear increase with increasing electric field strength, which confirmed the strong interaction among PANI/HNT particles.

(5) $$G\left(t\right)≅G^{\prime }\left(\omega \right)-0.560G^{\prime \prime }\left(\frac{\omega }{2}\right)+0.200G^{\prime \prime }\left(\omega \right)$$

In addition, Table 1 summarizes fabrication method, clay content and slope of dynamic yield stress of most electro-responsive polymer/clay nanocomposites covered in this review. The slope of dynamic yield stress of the ER fluids ranged from 1.2 to 2, implying that the mechanism seems to be dependent on not only different materials but also different fabrication methods.

**Table 1:** Summary of electro-responsive polymer/clay nanocomposites with fabrication method, clay content and slope of dynamic yield stress.

Polymer/Clay Nanocomposites	Fabrication Method	Clay Content (wt %)	Slope of Dynamic Yield Stress	Reference
PANI/MMT	*In-situ* polymerization	0.75–3	-	[41]
PEA/MMT	*In-situ* polymerization	0.5–5	-	[42]
PUA/MMT	Suspension polymerization	5	-	[47]
SAN/MMT	Emulsion polymerization	4.76	-	[49]
PANI/OMMT	Pickering emulsion polymerization	-	1.5	[52]
PIN/OMMT	*In-situ* polymerization	5.5–12.1	-	[81]
PIN/kaolinite	cationic radical polymerization	15–78	-	[80]
PANI/HNT	*In-situ* polymerization	-	2	[45]
PPy/HNT	*In-situ* polymerization	-	1.5	[68]
PANI/PAL	*In-situ* polymerization	-	2	[46]
PS/laponite	Pickering emulsion polymerization	8.85	1.2	[53]

## 4. Conclusions

Various electric stimuli-responsive polymer-clay nanocomposites synthesized by a range of methods were reviewed. SEM, TEM and XRD confirmed their successful intercalation. The thermal stability of the conducting polymer chain was also enhanced due to the shielding role of the clay. In addition, the conducting polymer/clay nanocomposite-based ER fluids also found showed excellent ER behaviors and follow the previously proposed CCJ model.
